# Fully automated pixel-wise myocardial blood flow quantification with first-pass perfusion CMR

**DOI:** 10.1186/1532-429X-18-S1-O87

**Published:** 2016-01-27

**Authors:** Li-Yueh Hsu, Matthew Jacobs, Mitchel Benovoy, Hannah M Conn, Andrew E Arai

**Affiliations:** 1 grid.94365.3d0000000122975165National Heart Lung and Blood Institute, National Institutes of Health, Bethesda, MD USA; 2 grid.39936.360000000121746686Department of Electrical Engineering and Computer Science, Catholic University of America, Washington, DC USA; 3 grid.183158.60000000404353292Department of Biomedical Engineering, Polytechnique Montreal, Montreal, QC Canada

## Background

Fully quantitative CMR perfusion pixel maps have been validated with microsphere myocardial blood flow (MBF) measurements and shown potential clinical applications. However, the process of quantification can be time-consuming. We developed a fully automated image processing method to generate MBF pixel maps from the first-pass CMR perfusion images.

## Methods

Rest and adenosine stress perfusion imaging was performed on 17 normal volunteers. A saturation recovery SSFP dual-sequence technique was used to acquire three myocardial slices and an arterial input function (AIF) image series. A proton-density weighted image was acquired at the beginning of each series. Fully quantitative perfusion pixel maps were generated by an automated processing method, including non-rigid motion correction, surface-coil intensity correction, AIF and myocardial signal and contrast timing detection, and model constrained deconvolution. The results of the automatically generated MBF pixel maps were compared with manual quantification using an 18-segment model.

## Results

**Figure 1:**
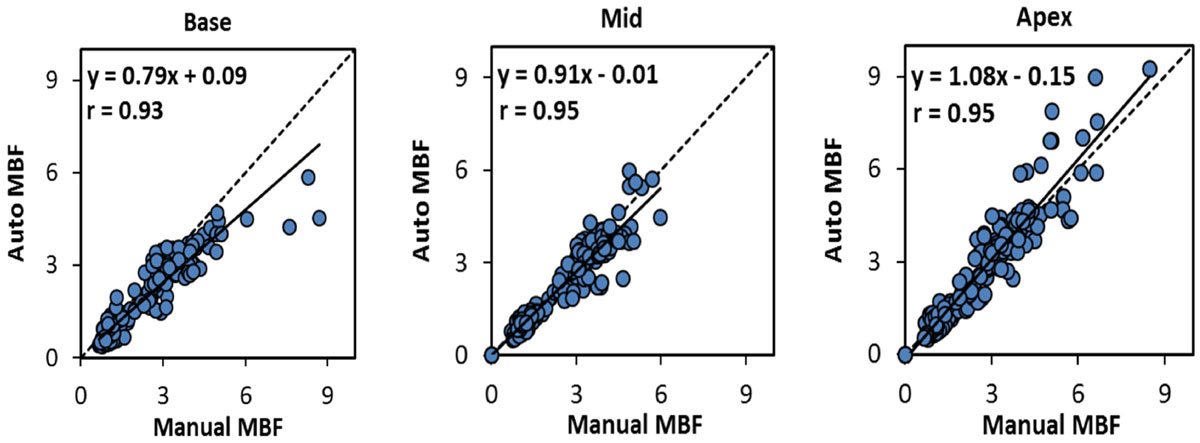
Figure 1

MBF was compared in 612 segments from 102 perfusion image series. There was an excellent Pearson's correlation between the automated and manual perfusion pixel maps in all slice locations (r = 0.93, 0.95, and 0.95, figure-1). Bland-Altman analysis showed no significant bias between the two methods (bias -0.20 ml/g/min).

## Conclusions

We present a fully automated pipeline for processing fully quantitative MBF maps from CMR perfusion images. Our results show the automatically generated perfusion pixel maps have a high degree of agreement compared with manual quantification.

